# Single‐cell transcriptomic analysis of PB and BM NK cells from severe aplastic anaemia patients

**DOI:** 10.1002/ctm2.1092

**Published:** 2022-12-05

**Authors:** Chunyan Liu, Yingying Chen, Dan Lu, Bingnan Liu, Tian Zhang, Ling Deng, Zixuan Liu, Congwei Zhong, Rong Fu

**Affiliations:** ^1^ Department of Hematology Tianjin Medical University General Hospital Tianjin China

**Keywords:** aplastic anaemia, cytokine‐induced memory‐like NK cell, natural killer cells, single‐cell RNA sequencing

Dear Editor,

In recently, we reported the important contribution of nature killer (NK) cells in the pathogenesis of severe aplastic anaemia (SAA). Our pilot research indicated that NK cell number and function were abnormal in SAA patients,^1^ and we also identified several proteins associated with NK cell dysfunction by proteomic analysis.^2^ To further explore the subgroups of NK cells that may be implicated in the pathogenesis of SAA and find the differences between bone marrow NK cells (BM‐NK) and peripheral blood NK cells (PB‐NK), we conducted single‐cell RNA sequencing (scRNA‐seq) on BM‐NK and PB‐NK of SAA patients and healthy volunteers (HC).

In keeping with our preceding studies,^1^ the percentage of BM‐NK was higher than that PB‐NK in both HC and SAA, and the percentage of PB‐NK and BM‐NK were both markedly lower in the SAA than in the HC (Figure S1). A total of 9280 cells (6134‐13100) from each sample were sequenced, averaging 1876 genes analyzed per cell. After Harmony integration and Scanpy filtering, 97424 cells were included for further analysis, and 20 clusters were obtained from the initial UMAP (Unifrom Manifold Approximation and Projection) and Louvain clustering (Figure S2). We removed the non‐NK cell clusters (Table S1) and finally obtained eight groups of NK cells (Figure 1A). These eight groups of NK cells were detected in all samples and passed quality control (Figure S3). We didn't detect NCAM (CD56) in the dataset and used CD7/KLRD1/KLRF1/NGK7/GNLY as specific NK cell markers.^3^ As expected, all these eight clusters expressed high levels of NK cell‐specific markers (Figure 1B). Referring to the previous literature of NK cell scRNA‐seq,^3^, ^4^, ^5^, ^6^ combined with the characteristic marker genes of each cluster, we named these eight NK clusters as transitional NK, adaptive NK, CD56^bright^ NK, mature NK, cytokine‐induced memory‐like (CIML) NK1(normal CIML NK), CIML NK2(active CIML NK), active NK and terminal NK (Table 1, Figure 1C,D, Figure S4). The cell number and the proportion of each NK cell subsets are shown Figure 1E and Table S3. We used RNA rate analysis^7^ to describe the differentiation trajectory of NK cells (Figure 1F) and found that terminal NK and CIML NK had unique transcriptional characteristics. CIML NK2 maybe derive from CIML NK1, and SCENIC analysis^8^ also showed that these two clusters had similar regulons (Figure 1G). What's more, the results showed that CD56^bright^ NK was in the early stage of NK cell differentiation, and transitional NK was a mixture of the intermediate process of NK differentiation, which is the transitional stage of CD56^bright^ NK, adaptive NK and active NK to mature NK.

**FIGURE 1:**
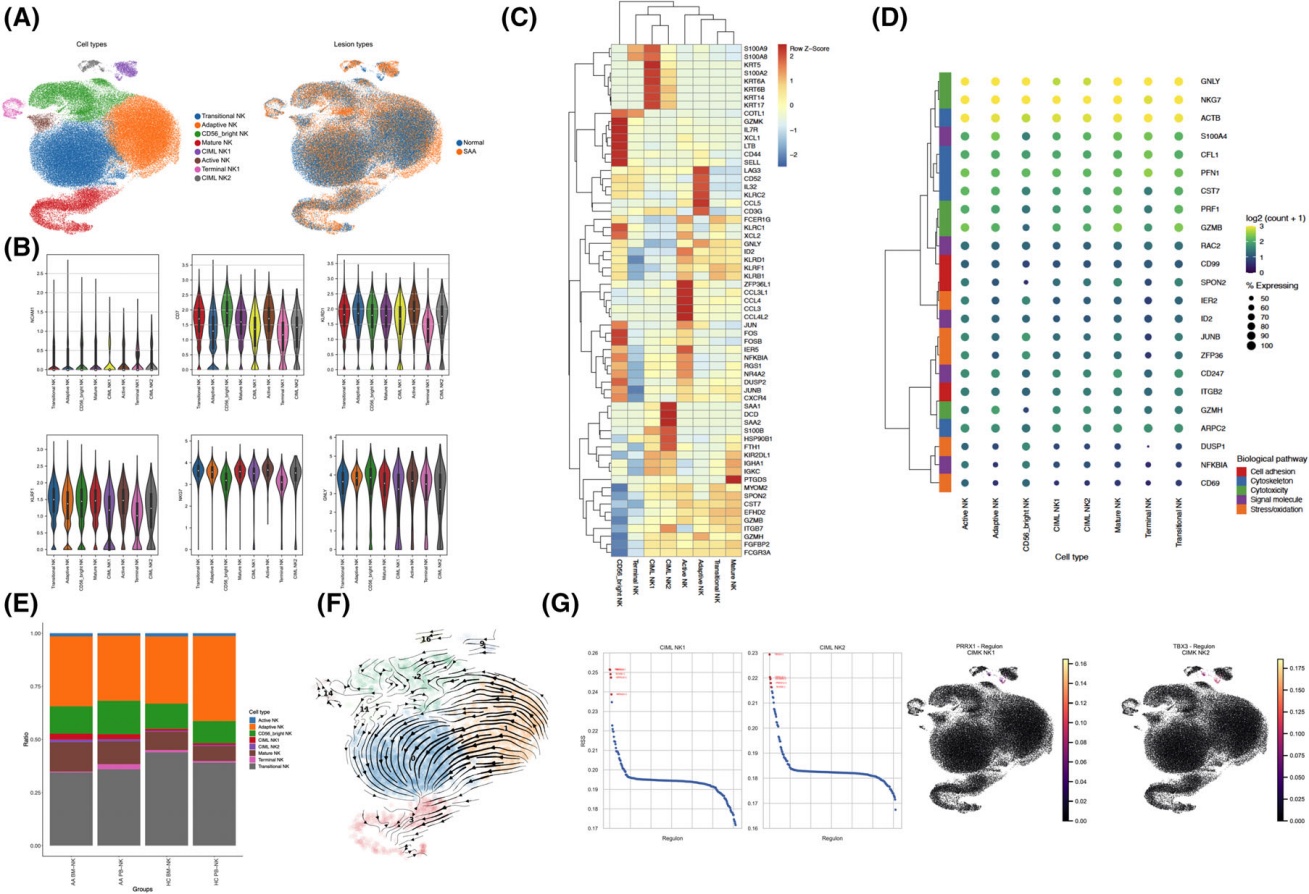
Overview of eight types of nature killer (NK) cells. (A) UMAP of 76156 cells from eight types of NK cells (left) and the corresponding lesion type of their donors (right). (B) Violin plot showing the expression level of six markers (NCAM1, CD7, KLRD1, KLRF1, NKG7 and GNLY) in eight types of NK cells. (C) Heatmap displaying the expression level of 63 target genes in eight types of NK cells. (D) Visualizing expression of genes associated with biological pathways. A dot plot showing the log‐transformed expression values of marker genes (y‐axis) from five subsets (labelled with different colours) across eight types of NK cells (x‐axis). (E) Stacked bar plots showing the percentage of different NK cells in four groups, from left to right, AA‐BM‐NK, AA‐PB‐NK, HC‐BM‐NK and HC‐PB‐NK. (F) RNA velocity unveils the dynamics of differentiation of eight types of NK cells. Terminal NK and cytokine‐induced memory‐like (CIML) had unique transcriptional characteristics, among which CIML NK2 was transformed from CIML NK1. (G) TFs (transcription factors) were identified by SCENIC across two CIML NK cells. Left: Relative‐specific scores (RSS) plot of TFs in two CIML NK cells. Right: Visualizing the TF with the highest RSS in two CIML NK cells on UMAP plot. A high RSS value indicates high correlation between TF and cells.

**TABLE 1:** NK cell population and nomenclature

Cluster	Name	Flow cytometric marker	Characteristic marker genes
Cluster 0	Transitional NK	–	TMIGD2, down‐regulated marker genes of CD56^bright^ NK, up‐regulated genes of CD56^dim^ NK
Cluster 1	Adaptive NK	CD56^dim^CD16^+^CD57^‐^	CXCR4, CD3D/3E/3G, KLRC2, CD52, IL32, CCL5, VIM, IGHA1, IGKC, LAG3
Cluster 2	CD56^bright^ NK	CD56^bright^CD16^‐^	IL7R,SELL, KLRC1, GZMK, LTB, COLT1, RGS1, XCL1, DUSP1, FOS, JUN, JUNB
Cluster 3	Mature NK	CD56^dim^CD16^+^CD57^+^	GNLY, NKG7, GZMB, PRF1, FGFBP2, CST7, KLRF1, KLRB1
Cluster 9	CIML NK1	CD56^dim^CD16^+^	GZMH, MYOM2, FGFBP2, IL2RB, FCGR3A, KIR2DL1, CD3G, S100 and keratin families
Cluster 11	Active NK	CD56^dim^CD16^+^CD57^‐^	CXCR4, CCL4, CCL3, CCL4L2, CCL3L1,CD69, XCL2, JUN, KLRB1, KLRD1, FOS, NR4A2, NFBKIA
Cluster 14	Terminal NK	CD56^dim^CD16^+^CD57^+^	ZEB2, HAVCR2, CX3CR1, high expression of microtubule‐ and cytoskeleton‐related genes
Cluster 16	CIML NK2	CD56^dim^CD16^+^	Same as CIML NK1, in addition, highly expressed S100B, SAA1, SAA2, FTH1, HSP90, PTGDS

After identifying the subgroup of NK cells, we analyzed the BM‐NK and PB‐NK transcriptomic changes in HC, identified 417 differentially expressed genes (DEGs) (Figure 2A). Note that 88.97% (371/417) of these DEGs were identified in only one cell types, and most of these genes were the characteristic genes of each cluster. Except for terminal NK cells, the gene expression of NK subsets in BM (bone marrow) and PB (peripheral blood) was basically similar, indicating that the function of BM‐NK and PB‐NK were consistent, including immunomodulatory and cytotoxic functions (Figure 2B). Terminal NK has active DNA replication (Figure 2C). Interestingly, PB terminal NK cells had a stronger cytotoxic function and cell killing/ NK cell mediated cytotoxicity pathways were up‐regulated, while BM terminal NK cells had stronger immune regulatory function, and immune‐related pathways were up‐regulated (Figure 2D,E). The results indicated that terminal NK cells with different localization have different functional tendencies.

**FIGURE 2:**
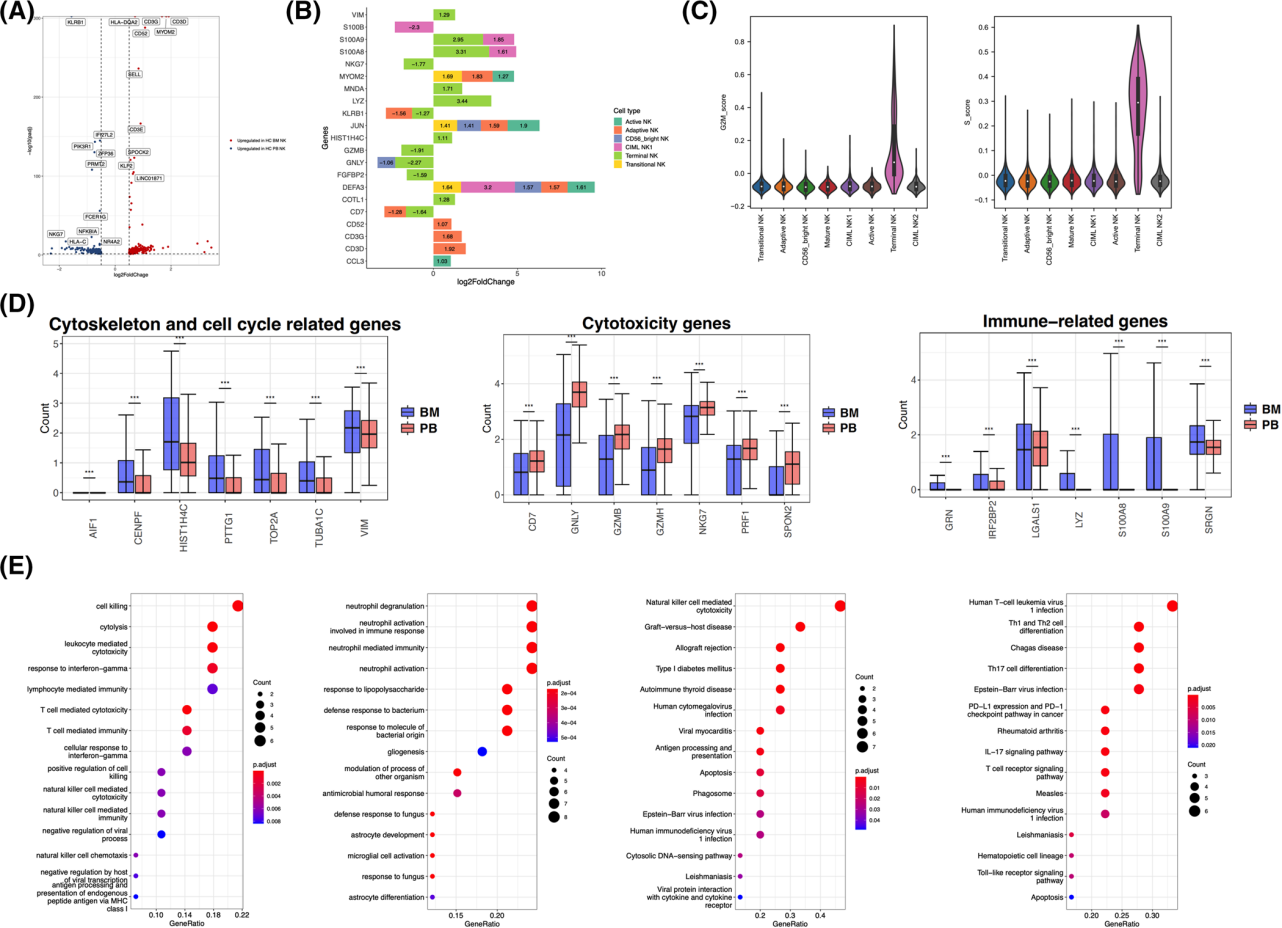
Comparison of PB‐nature killer (NK) and BM‐NK cells in healthy volunteers (HC). (A) Volcano plot showing differential expression genes (DEGs) across NK cells in BM and PB samples collected from HCs. A total of 417 DEGs were identified in HC BM‐NK versus HC PB‐NK (Wilcoxon rank sum test, absolute log2FC ≥ .5, Bonferroni adjusted *p* value < .05), including 234 up‐regulated genes in BM‐NK and 183 up‐regulated genes in PB‐NK. (B) LogFC bar graph of DEGs identified in NK cells by comparing HC‐BM cells with HC‐PB cells. Genes with log2FC > .5 indicate that genes are up‐regulated in HC‐BM and vice versa in HC‐PB. (C) Violin plots showing the cell cycle score for each type of NK cells. Left: G2M phase. Right: S phase. (D) Expression level of specific genes in terminal NK cells across BM and PB samples. Left: cytoskeleton and cell cycle‐related genes. Middle: cytotoxicity genes. Right: immune‐related gene. (E) Functional enrichment analysis of DEGs in terminal NK cells. From left to right, GO (Gene Ontology) enrichment analysis of the up‐regulated genes in the PB terminal NK, GO enrichment analysis of the up‐regulated genes in the BM terminal NK, KEGG (Kyoto Encyclopedia of Genes and Genomes) enrichment analysis of the up‐regulated genes in the PB terminal NK and KEGG enrichment analysis of the up‐regulated genes in the BM terminal NK. The color of the bubble indicates the adjusted *p*‐value of the GO terms or pathways and the size of the bubble signifies the number of genes associated with a term.

Next, we identified 245 DEGs in BM‐NK and 126 DEGs in PB‐NK between SAA and HC (Figure 3A). Of these DEGs, 44.9% and 47.6% DEGs appeared in multiple subgroups, and 58.73% of DEGs in PB‐NK cells were consistent with BM‐NK (Figure 3B), suggesting that the same NK subgroup in different position (PB or BM) and different NK subgroups in the same position had partial similar functional changes under pathological conditions, especially in CD56^bright^ NK, transitional NK, adaptive NK and mature NK (Figure S5). We found that, in SAA patients, both BM and PB‐NK played a stronger immunomodulatory role, while the cytotoxic function was down‐regulated, which may be disease‐related functional depletion. What's more, in SAA BM‐NK, we detected more up‐regulation of immunomodulatory genes and more down‐regulation of killing related genes and found several transcription factors and their ligands were up‐regulated (Figure 3C,D). Therefore, we believe that BM‐NK was more sensitive than PB‐NK in the disease state.

**FIGURE 3:**
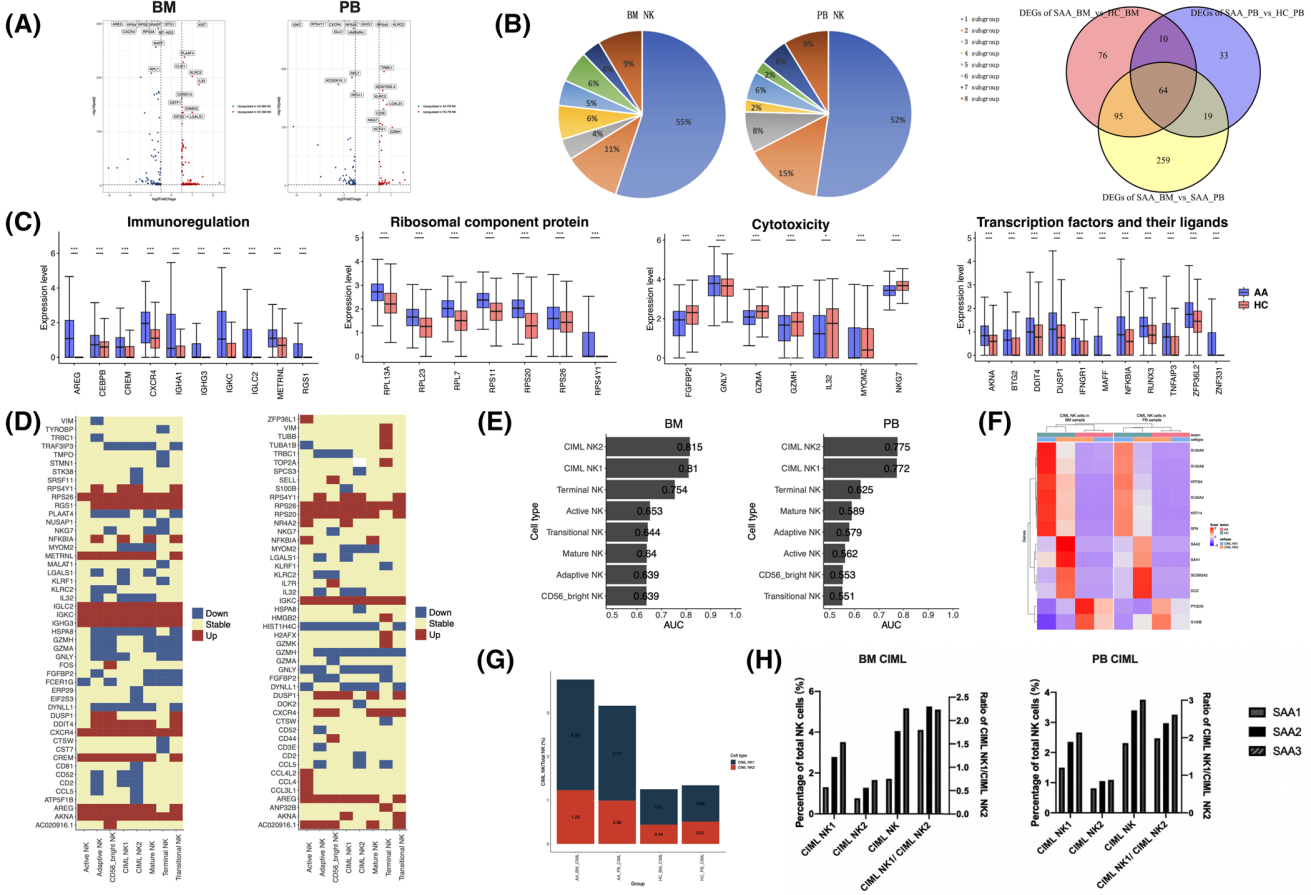
Comparison of PB‐nature killer (NK) and BM‐NK cells between severe aplastic anaemia (SAA) and HC. (A) Volcano plot showing differentially expressed genes (DEGs) of BM‐NK cells (left) and PB‐NK cells (right) between SAA and HC. A total of 245 and 126 DEGs were identified in BM‐NK and PB‐NK between SAA and HC (Wilcoxon rank sum test, absolute log2FC ≥ .5, Bonferroni adjusted *p* value < .05), including 141 up‐regulated genes and 104 down‐regulated genes in BM‐NK of SAA patients, 66 up‐regulated genes and 60 down‐regulated genes in PB‐NK of SAA patients. (B) Summary of DEGs between SAA and HC. Left‐Middle: Pie charts showing the distribution of differential expression genes (DEGs) isolated from BM (left) and PB (middle) samples across eight types of NK cells. Venn diagram showing the differential expressed genes (DEGs) detected by pair‐wise comparison at three groups. (C) Box plots showing expression of the different function genes in SAA and HC. From left to right: immunoregulation‐related genes, ribosomal component protein coding genes, cytotoxicity‐related genes, transcription factors and their ligands genes. (D) Heatmap displaying the expression level of some DEGs in eight types of BM‐NK cells (left) and PB‐NK cells (right) based on significance and logFC values. Pair‐wise comparisons of each type of NK cell between HC group and AA group in BM and PB samples were peformed to calculate the significance and logFC values of genes. (E) Prioritization of cell types responsive to SAA with Augur. Left: AUC value of NK cells collected from BM. Right: AUC value of NK cells collected from PB. A high AUC value indicates the high sensitivity of the cells responding to changes in the SAA. Both cytokine‐induced memory‐like (CIML) NK1 and CIML NK2 contributed significantly to disease phenotype in BM and PB (BM: AUC .815 and .810; PB: AUC: .725 and .772). (F) Heat map of CMIL NK cell‐specific gene expression profiles in PB‐NK and BM‐NK of SAA patients/ HCs. (G) The heatmap showing the expression profiles of specific genes between the HC group and AA group in BM and PB samples. (H) The percentage of two CIML NK cells among four groups. (I) BarPlot showing the different percentages of two CIML NK cells collected from SAA patients. Left: BM CIML NK cells. Right: PB CIML NK cells

Finally, through Augur^9^ analysis, we found that CIML NK1 and CIML NK2 were the most significantly cell clusters, which were contributed to disease phenotype in both BM and PB (BM: AUC (Area Under Curve) .815 and .810; PB: AUC: .725 and .772) (Figure 3E). Both in PB and BM, the percentage of CIML NK cells of SAA patients was higher than that in HC, but the ratio of active CIML NK/total CIML NK was decreased (Figure 3F). At the same time, the characteristic genes of BM and PB CIML NK cells in SAA were down‐regulated and the pathways were enriched in cytokine stimulation and immune response (Figure 3G). Further analysis showed that among these three SAA patients, those with a higher proportion of CIML NK cells and a higher CIML NK1/CIML NK2 ratio had a better prognosis (Figure 3H, Table S4). So, we speculate that the increase proportion of CIML is a protective response in SAA, while the decrease in proportion and function of active CIML NK is a compensatory‐related exhaustion. CIML NK cells may be reliable predictors of SAA treatment outcome. Exploration of the role of CIML NK in the occurrence and development of SAA will further clarify the immune pathogenesis of SAA and provide ideas for new therapeutic methods.

In conclusion, scRNA‐seq can more accurately reflect the function of NK subsets and enrich our understanding of NK cells. BM‐NK and PB‐NK cells have similar functions, and BM‐NK cells are more sensitive in the disease state. Further investigation is warranted to examine whether CIML NK cells are associated with treatment response. Additional samples and further analysis are needed to validate the results of different NK subsets.

## CONFLICT OF INTEREST

The authors declare that they have no competing interests.

## Supporting information

Supporting Information
