# Supplementary material for: Single‐cell transcriptomic analysis of PB and BM NK cells from severe aplastic anaemia patients
Source: Clin Transl Med. 2022 Dec 5;12(12):e1092. doi: 10.1002/ctm2.1092 (PMC9722964; doi:10.1002/ctm2.1092)
Supplement: Supplementary file 1 — Supporting Information [file CTM2-12-e1092-s001.docx]

**SUPPORTING MATERIALS**

6 Supporting figures

5 Supporting tables

Materials & Methods

The naming rules of each NK cell subgroup

**6 Supporting figures**


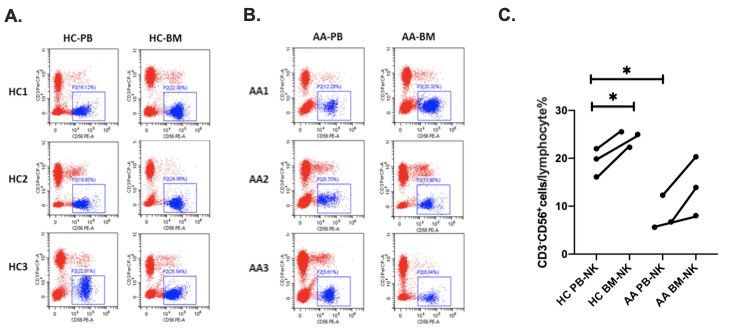


Figure S1. The proportion of NK cells (CD3^-^CD56^+^) in BM and PB was determined by flow cytometry. A. The results showed that in the HC group, NK cells accounted for 19.53±1.637% and 24.46±2.048% of the lymphocytes in PB and BM, respectively. B. In the SAA group, the proportion were 8.41±3.438% and 13.11±5.041%. C. In both groups, the ratio of NK cells was higher in BM than in PB (*p*=0.0234, *p*=0.0778).The ratios of NK cells in PB and BM were lower in the SAA group than in the HC group (*p*=0.0144, *p*= 0.0506).


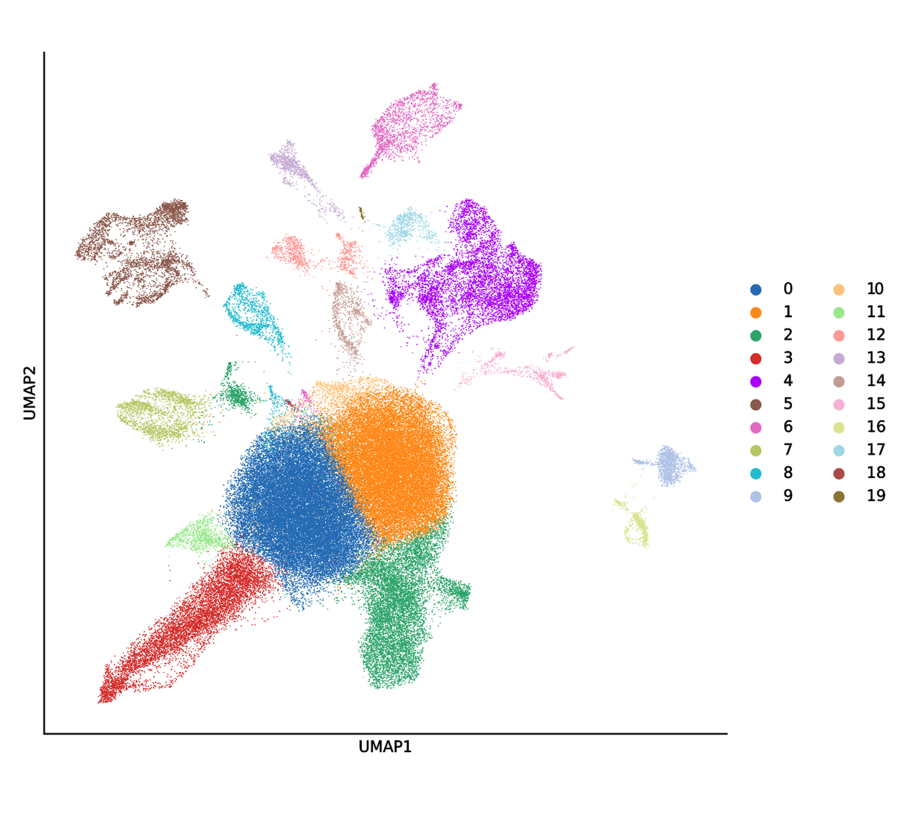


Figure S2. By UMAP and Louvain clustering, 97424 cells were shown as 20 groups of cells. According to the gene expression patterns of different clusters and specific marker genes, we named each group of cells as: cluster0-Transitional NK, cluster1-Adaptive NK, cluster2- CD56bright NK, cluster3- Mature NK, cluster4-Granulocyte, cluster5-B cell1, cluster6-Megakaryocyte1, cluster7-T cell1, cluster8-Red blood cell, cluster9-CIML NK1, cluster10-T cell2, Cluster11-Active NK, cluster12-B cell2, cluster13- Dendritic cell, cluster14-Terminal NK, cluster15-Megakaryocyte2, clustre16-CIML NK2, cluster17-Monocyte, cluster18-Quiescence or senescence cells, cluster19- Hematopoietic stem and progenitor cell.

Figure S3. Quality control of eight types of NK cells. Most of the sequenced cells had more than 5000 unique molecular identifiers (left) and at least 1500 genes related to cell barcodes (middle). Most cells were highly viable because mitochondrial transcription accounted for <7% of total gene expression (right).

Figure S4. The expression of the top 2 markers which distinguish each cluster. Expression is color coded from blue (low) to red (high) and cells positively expressing a marker are brought toward the front of the plot.

Figure S5. Functional enrichment analysis of DEGs between SAA and HC. A. GO enrichment of DEGs among SAA BM-NK cells and HC BM-NK cells (left) , GO enrichment of DEGs among SAA PB-NK cells and HC PB-NK cells (right). B. KEGG enrichment of DEGs among SAA BM-NK cells and HC BM-NK cells (left), KEGG enrichment of DEGs among SAA PB-NK cells and HC PB-NK cells (right). The color of the bubble indicates the adj.P-value of the GO terms or pathways and the size of the bubble signifies the number of genes associated with a term.


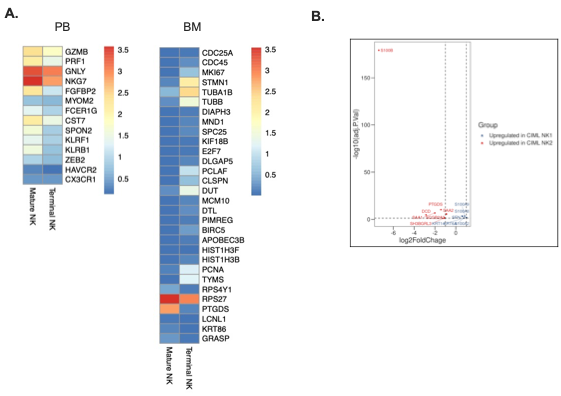


Figure S6. DEGs of mature NK and terminal NK, CIML NK1 and CIML NK2 in HCs. A. Heat map of DEGs expression profiles in PB-NK (left) and BM-NK (right) of HCs. B. Volcano plot showing DEGs of CIML NK1 and CIML NK2 (Wilcoxon rank sum test, absolute log2FC ≥ 1, Bonferroni adjusted p value <0.05) in HCs.

**5 Supporting tables**

Supplementary Table 1. Basic information and treatment information of 3 SAA patients.

|  | Sex | Age | CD4/CD8 | Hb  (g/L) | WBC  (×10^9^/L) | PLT  (×10^9^/L) | RBC  (×10^12^/L) | ANC  (×10^9^/L) | The time between IST and diagnosis | curative effect |
| --- | --- | --- | --- | --- | --- | --- | --- | --- | --- | --- |
| SAA1 | F | 40 | 0.23 | 60 | 2.01 | 39 | 1.97 | 0.64 | 28d | NR |
| SAA2 | F | 51 | 0.97 | 31 | 1.14 | 38 | 0.99 | 0.16 | 30d | PR |
| SAA3 | M | 56 | 1.21 | 77 | 1.05 | 27 | 2.73 | 0.04 | 11d | CR |

Supplementary Table 2. The Percentage and markers of non-NK cell clusters

| Cluster name | Cluster number | Percentage（%） | Marker or characteristic |
| --- | --- | --- | --- |
| Granulocyte | 4 | 7.14 | CD24/ AZU1/ MPO |
| B cell | 5、12 | 4.93 | CD19/ CD79a |
| Megakaryocyte | 6、15 | 3.04 | PF4/ GP9/ ITGA2B/ PPBP |
| T cell | 7、10 | 3.33 | CD3G/ CD3D/ CD3E/ IL7R/ CCR7/ TCF7 |
| Red blood cell | 8 | 1.65 | HBA1/ BLVRB/ GATA1 |
| Dendritic cell | 13 | 0.91 | CD86/ CLEC7A |
| Monocyte | 17 | 0.67 | CD14/ CXCR1 |
| Quiescence or senescence cells | 18 | 0.60 | RB1CC1/ GABARAPL11，mitochondrial transcription ratio, unique molecular identifiers, and number of expressed genes were lower than other clusters, only detected in the BM sample of SAA2 |
| Hematopoietic stem and progenitor cell | 19 | 0.065 | GATA2/ SPINK2 |

Supplementary Table 3. Cell number and proportion of NK cell subsets in different groups.

| Cell number of NK cell subsets in different groups | | | | | |
| --- | --- | --- | --- | --- | --- |
|  | HC PB-NK | HC BM-NK | AA PB-NK | AA BM-NK | Significant difference |
| transitional NK | 2899.667±917.643 | 1798±1150.144 | 2463.333±1288.463 | 2415.667±711.716 | *#￥ |
| adaptive NK | 2969±1581.351 | 1293.667±380.713 | 2101.333±932.903 | 2309.667±719.173 | *#￥ |
| CD56 bright | 774±117.286 | 485.667±279.038 | 1080±559.74 | 902±100.18 | *#￥ |
| mature NK | 525.667±314.238 | 351±321.565 | 741±639.516 | 969±842.748 | *#￥ |
| CIML NK1 | 61±51.391 | 38.667±31.342 | 157.667±84.008 | 193.667±123.662 | *#￥ |
| active NK | 100±34.044 | 63.333±52.548 | 78.333±21.032 | 105.333±84.619 | ￥ |
| terminal NK | 53±9.849 | 42±6.928 | 163.333±151.19 | 29±18.682 | ￥ |
| CIML NK2 | 37.667±31.644 | 21.333±18.448 | 70±32.047 | 92.333±54.308 | #￥ |
| Proportion of NK cell subsets in different groups. | | | | | |
|  | HC PB-NK | HC BM-NK | AA PB-NK | AA BM-NK | Significant difference |
| transitional NK | 0.394±0.101 | 0.411±0.105 | 0.339±0.070 | 0.350±0.045 | *#￥ |
| adaptive NK | 0.394±0.157 | 0.362±0.141 | 0.302±0.039 | 0.332±0.043 | *#￥ |
| CD56 bright | 0.104±0.007 | 0.117±0.007 | 0.199±0.145 | 0.142±0.055 | *#￥ |
| mature NK | 0.071±0.038 | 0.071±0.037 | 0.091±0.063 | 0.118±0.080 | *#￥ |
| CIML NK1 | 0.008±0.005 | 0.008±0.004 | 0.022±0.005 | 0.025±0.009 | *#￥ |
| active NK | 0.014±0.005 | 0.015±0.005 | 0.014±0.008 | 0.015±0.008 | ￥ |
| terminal NK | 0.007±0.001 | 0.012±0.004 | 0.024±0.014 | 0.005±0.004 | ￥ |
| CIML NK2 | 0.005±0.003 | 0.004±0.003 | 0.010±0.001 | 0.012±0.04 | #￥ |

(Significant difference between different groups: p＜0.05，* HC PB-NK vs. HC BM-NK, # HC BM-NK vs. AA BM-NK, $ HC PB-NK vs. AA PB-NK)

Supplementary Table 4. The proportion of CIML in three SAA patients.

|  | curative effect | BM | | | | PB | | | | |
| --- | --- | --- | --- | --- | --- | --- | --- | --- | --- | --- |
|  |  | CIML NK1 | CMIL NK2 | Total CIML NK | CIML NK1/CIML NK2 | CIML NK1 | CMIL NK2 | Total CIML NK | CIML NK1/CIML NK2 | |
| SAA1 | NR | 1.30% | 0.76% | 2.06% | 1.71 | 1.5% | 0.81% | 2.31% | | 1.85 |
| SAA2 | PR | 2.78% | 1.27% | 4.05% | 2.19 | 2.35% | 1.05% | 3.4% | | 2.24 |
| SAA3 | CR | 3.51% | 1.65% | 5.16% | 2.13 | 2.66% | 1.09% | 3.75% | | 2.44 |

Supplementary Table 5. Summary of NK cell population and characteristics in the NK cell scRNA-Seq literature.
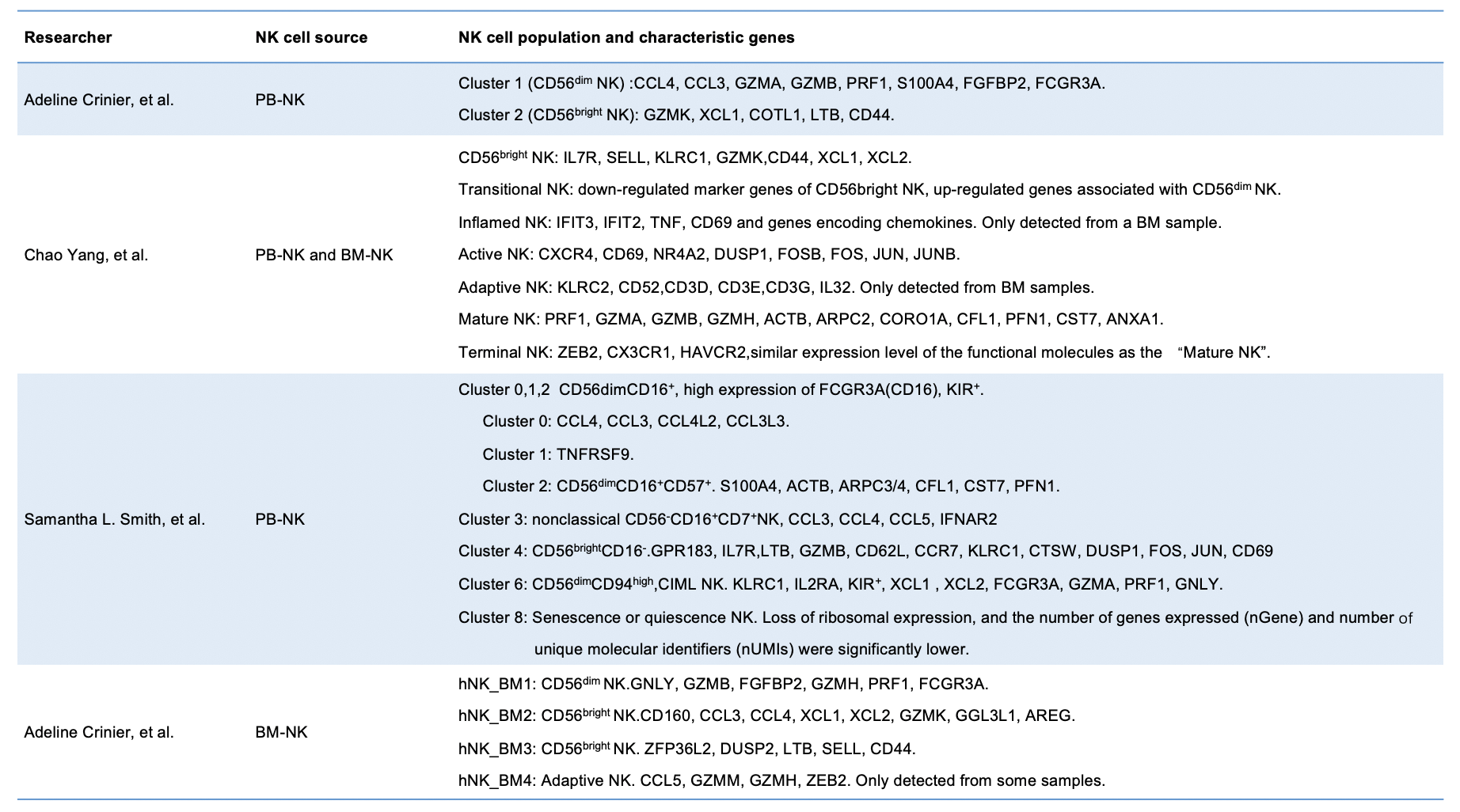


**Materials and Methods**

**1.Patients and clinical samples**

Three treatment-naïve SAA patients, one man and two women with a median age of 51 years (40-57) admitted to the General Hospital of Tianjin Medical University between June 2020 and October 2020 (SAA group), were enrolled in this study. The diagnostic and treatment outcome evaluation of all three patients were carried out according to relevant guidelines. In all cases, other diseases that may cause pancytopenia such as paroxysmal nocturnal hemoglobinuria, myelodysplastic syndrome, mycosis fungoides, and Fanconi’s anemia were ruled out. Iron overload, malignant tumors, autoimmune diseases, viral infections (e.g., cytomegalovirus, Epstein-Barr virus, hepatitis virus), and other infections relevant diseases were ruled out.

Due to the rarity of SAA samples, we sent them for scRNA-seq in three batches. Once the diagnosis of a SAA patient was confirmed, BM and PB sample were obtained from the patient before starting treatment. When a suitable AA patient is available for sampling, PB and BM samples of a healthy volunteer were collected simultaneously. So, the controls were not well matched for age and sex. Three healthy volunteers (HC group) were all women, with a median age of 38 years (35-53 years). Each batch of specimens was sampled at the same time, processed and submitted for scRNA-seq to minimize experimental errors.

All patients received intensive immunotherapy (antithymocyte globulin [ATG] + cyclosporin) and hematopoietic therapy (granulocyte-colony-stimulating factor, erythropoietin, thrombopoietin/eltrombopag). One nonresponder (SAA1) died of infection 12 months after treatment, one patient (SAA2) was weaned off blood transfusion, and one patient (SAA3) had completely normal blood tests. The median follow-up time was 14 months (12-16). The general information and treatment data of these three SAA patients were shown in Table S1. The protocol was approved by the Ethics Committee of the General Hospital of Tianjin Medical University. Each subject or his/her family signed the informed consent.

**2.Sample collection and NK cell sorting**

20 mL of heparin anticoagulant PB and BM from SAA patients or HCs were collected to separate mononuclear cells with lymphocyte separation medium (Amersham Bioscience, UK) under sterile conditions. Mononuclear cells were washed with sterile phosphate-buffered saline, The percentage of NK cells was determined by flow cytometry (Calibur, BD Biosciences, USA) using CD3-PerCP (347344, BD Biosciences, USA), CD56-PE (555516, BD Biosciences, USA). The remaining cells were suspended in 1ml of sterile magnetic bead sorting buffer and counted. Negative magnetic beads (130-092-657, Miltenyi Biotec, USA) were used to sort NK cells, and the NK cells was washed with sterile phosphate-buffered saline, suspended, and counted. The cell suspension was adjusted to 300-600 cells/μL, and cell viability was analyzed with Countstar Rigel S2. Sequencing analysis was carried out after passing quality inspection.

**3. Single-cell RNA sequencing**

***Cell capture and cDNA synthesis*** Cell capture was done with the Single Cell 3′ Library and Gel Bead Kit v3.1(10x Genomics, 1000121). cDNA was synthesized with the Chromium Single Cell G Chip Kit (10x Genomics, 1000120). The cell suspension (300-600 living cells per microliter determined by Count Star) was loaded onto the Chromium single cell controller (10x Genomics) to generate single-cell gel beads in an emulsion according to the manufacturer’s protocol. In brief, single cells were suspended in phosphate-buffered saline containing 0.04% BSA. Approximately 20,000 cells were added to each channel, and the target cells recovered were estimated at approximately 10,000 cells. Captured cells were lysed, and the released RNAs were barcoded through reverse transcription in individual gel beads in emulsion. Reverse transcription was performed on an S1000TM Touch Thermal Cycler (Bio Rad) at 53°C for 45 min followed by 85°C for 5 min. The cDNA was then amplified by PCR and its quality assessed using an Agilent 4200 (performed by CapitalBio Technology, Beijing).

***Single cell RNA-Seq library preparation*** According to the manufacturer’s instructions, single-cell RNA-seq libraries were constructed using the Single Cell 3′ Library and Gel Bead Kit v3.1. The libraries were sequenced in an Illumina Novaseq6000 sequencer with a sequencing depth of at least 100,000 reads per cell with a paired-end 150-bp reading strategy (performed by CapitalBio Technology, Beijing).

**4.Single-cell RNA data preprocessing**

***Cell ranger pipeline*** Cell Ranger version 3.1.0 software (10x Genomics website, https://support.10xgenomics.com/single-cell-gene-expression/software/downloads/latest) was used to demultiplex the FASTQ reads and align them to the human transcriptome (GRch38, provided by 10x Genomics) with the default parameters. The output of this pipeline contains a digital gene-barcode matrix for each sample. Then, all the matrices were subjected to Scanpy version 1.6.0 for further data processing. Cells with more than 6,000 or less than 200 detected genes as well as those with a mitochondrial transcription ratio >15% were discarded. Cell doublets (occasional pairs of cells that are not dissociated during sample preparation) were identified using the scrublet tool and then discarded. Normalization was performed using the Sctransform function provided by Seurat package. Then, batch effects derived from technical and biological covariates, including the batch and harvested time, were corrected using harmonypy version 0.0.5. Next, the graph-based clustering algorithm of Louvain after computing the nearest neighbor graph was used to partition the cells. The resulting clusters were visualized in a 2-D embedding produced by uniform approximation and projection (UMAP, version 0.4.6). Finally, the resulting coordinates of UMAP and cluster tags for each cell were assigned to the expression matrix for downstream analysis. Cell cycle stage was scored using the score_genes_cell_cycle() function.

***Estimation of the cell proportion*** The detected number of each cell type was modeled as a random count variable using a Poisson process. The rate of detection was modeled by providing the total number of captured cells in each sample as an offset variable, with the condition of each sample (health or disease) provided as a covariate.

***Differential expression analysis*** The empirical bayes linear model-based method of limma-voom was used to detect genes differentially expressed in each cell type between conditions. The discrete and continuous coefficients of the model were retrieved, *P* values were computed by performing the likelihood ratio test, and *q* values were separately evaluated for each cell subset comparison using the Benjamini-Hochberg correction. Genes with an expression |log2FoldChange|>0.5 and FDR<0.05 were considered differentially expressed.

***Cell type prioritization*** The cell types between conditions were ranked using Augur tool with default parameters. Briefly, the single cell matrix and cell type information were inputted and cells were ranked using the *calculate_auc()* function.

***Overrepresentation test for Gene Ontology (GO) and KEGG terms*** R packages “clusterProfiler” was used to perform biological function enrichment analysis. The top 15 GO terms including biological process (BP) were shown in the bubble chart. Benjamini-Hochberg (BH) method was carried out for multiple testing.

***Cell Cycle Phase*** Cell Cycle Phase was assigned by Seurat 3.0 and the same parameters were used.

***SCENIC analysis*** SCENIC was used to perform Gene regulatory network analysis with default parameters. Relative specific scores (RSS) of TFs were visualized by UMAP plot. Generally, a high RSS value indicates high correlation between TF and cells.

**5. Statistical Analysis**

The experimental data were analyzed using the statistical software GraphPad Prism 8.2.1(GraphPad Software, La Jolla, CA, USA). Quantitative data are presented as the mean±SD and qualitative data are presented as a percentage (%). Differences between 2 groups were analyzed using the independent sample t-test, paired sample t test or Mann–Whitney test. Multiple groups of data with a normal distribution and homogeneous variance were compared using the chi-squared test, multiple groups of data with a nonnormal distribution or nonhomogeneous variance were compared using the Kruskal–Wallis test. **p* < 0.05, ***p* < 0.01, ****p* < 0.00 1, and *****p* < 0.0001 were considered to be statistically significant.

**6. Data availability**

The RNA-seq data are deposed in GEO database under accession number GSE214207. Codes for bioinformatics analysis are freely available at https://www.ncbi.nlm.nih.gov/geo/query/acc.cgi?acc=GSE214207.

**The naming rules of each NK cell subgroup**

Although the populations of NK cells in different literatures are not the same^[3-6]^ (Table S5), we tried our best to find the consistent parts and combined with the characteristic marker genes of each cluster in our results, and named each population of NK cells according to their function and differentiation stage. The characteristics and marker genes of each subpopulation of NK cells are summarized in Table 1.

First, cluster 2 was identified as CD56^bright^ NK, which corresponded to CD56^bright^CD16^-^ cells in flow cytometry, a cluster of immature NK cells that may produce large amounts of cytokines with an immunomodulatory role. This group of cells is the most easily identified and the most consistent group of NK cells in various literatures. scRNA-seq showed high expression of IL-7RA (IL-7R), CD62L (SELL), NKG2A (KLRC1), GZMK, LTB, COLT1 and RGS1; low expression of FCGR3A (CD16), CD160 and B3GAT1 (CD57). We also found high expression of lymphatic chemokine (XCL1) and immediate-early gene (IEG) DUSP1, FOS, JUN and JUNB. All of these genes play an important role in the function regulation of NK cells.

Cluster 3 and Cluster 14 were mature NK and terminal NK, respectively. Although CD57 expression was not detected in our data set, we believe that these two clusters correspond to CD56^dim^CD16^+^CD57^+^ cells in flow cytometry and mainly play cytotoxic roles. Cluster 3 expressed high levels of granulysin (GNLY), NKG7, GZMB, perforin 1(PRF1), FGFBP2, CST7 and NK cell-surface receptors KLRF1 and KLRB1, indicating that this cluster is a mature cytotoxic NK cell with strong killing effects. Cluster14 was terminal NK, in which cells in the S and G2 phases were significantly increased, indicating active DNA replication and protein synthesis (Figure 2C). These two clusters had similar expression pattern of killing-related genes, in addition, terminal NK expressed high levels of microtubule- and cytoskeleton-related genes STMN1, TUBA1C, TUBB, DIAPH3 compared to mature NK (Figure 2E).  Although the expression levels of ZEB2, HAVCR2 (TIM3) and CX3CR1, which were important markers of mature NK cells ^[7, 8]^, were not as high as those of killing-related genes, they were still significantly higher than CD56bright NK. Meanwhile, terminal NK had unique transcription characteristics different from mature NK, such as high expression of cell cycle regulating gene MND1, SPC25, KIF18B, DLGAP5, E2F7 and division DNA replication– and repair–related genes PCLAF, CLSPN, DUT, MCM10, DTL histone subunit HIST1H3F, HIST1H3B, and cell proliferation–related genes PCNA and TYMS (Figure S6A). In addition, the ribosome-related genes RPS4Y1, RPS27, RPS14, RPS28 and RPS11 were depleted due to active DNA and protein synthesis. These unique transcription characteristics genes were enriched in the cell cycle, DNA replication and repair. Chao Yang et al. ^[3]^ also found in this cluster the histone methylation, chromosome maintenance, and telomere maintenance genes were enriched. Samantha L. Smith ^[5]^ found this cluster do not respond to IL-2 stimulation, which also proved that this population of cells functionally differentiated and mature, located in the terminal stage of development.

Cluster 11 was active NK, and Cluster 1 was adaptive NK. These two clusters expressed high levels of CXCR4, which is involved in the homing of NK cells to BM. Therefore, these two NK clusters had yet to differentiate into mature NK cells and represented to CD56^dim^CD16^+^CD57^-^ NK cells on flow cytometry. Active NK cells expressed high levels of chemokines CCL4, CCL3, CCL4L2, CCL3L1, and XCL2, all of which are closely related to the activation of NK killing, and of NK cell receptors KLRB1, KLRD1, KLRF1, and HAVCR2. We deduced that this cluster was similar to the chemokine-activated killers, such as CC-chemokine-activated killers (CHAK). Interestingly, as with CD56^bright^ NK, active NK expressed high levels of the early response genes/IEGs ZFP36L1, DUSP1, IER2, IER5, JUN, JUNB, FOS, and CD69, as well as transcription factors NFΚBIA, ID2, and NR4A2 (Figure 1E). Cluster 1 was adaptive NK, with high expression of KLRC2 (NKG2C), CD52, IL-32, CCL5, CD3D/3E/3G, VIM and low expression of EAT-2, SYK, PLZF (ZBTB16), FCER1G. Compared with mature NK, the expression of cytotoxicity-related genes (PRF1, GZMB, MYOM2) was lower, and the expression of ribosomal genes (RPS26, RPS12, RPS19, RPL6) and immunoglobulin-related genes (IGHA1, IGKC) was higher in this cluster. We deduced that despite lack of active gene transcription, adaptive NK cells were engaged in active protein synthesis to express and store functional proteins. In addition, the expression of LAG3, a novel immunotherapy target, was higher in adaptive NK than in other clusters.

Cluster 0 was transitional NK, with high expression of KLRF1, GZMA, GNLY and PFN1. It also expressed similar CD56^dim^-related genes (NKG7, GZMB, FCER1G, KLRB1, PRF1, SPON2), with high expression of antigen presentation related genes. In this cluster, the marker genes of CD56^bright^ NK were down-regulated, the genes associated with CD56^dim^ NK were up-regulated. Conventionally, NK cells are identified by cell surface expression of CD56 and are further divided into CD56^bright^ and CD56^dim^ NK cells. By RNA rate analysis ^[7]^ , we confirmed that CD56^bright^ NK cells are precursors of CD56^dim^ NK cells with a transitional population, which was consistent with previous studies ^[3]^ . Therefore, the gene expression and function patterns of transitional NK shared the characteristics of several clusters (CD56^bright^ NK, active NK, adaptive NK and mature NK. Moreover, the characteristic marker genes of transitional NK included transmembrane and immunoglobulin domain 2 (TMIGD2), a class of NK-regulatory genes.

Cluster 9 and Cluster 16 were CIML NK, with high expression of GZMH, MYOM2, FGFBP2, KIR2DL1, IL2RB, FCGR3A (CD16), CD3G. CIML NK is a newly discovered NK cell that produces more interferon -γ and GZMB when activated by cytokines in the stimulated state. These cells have an enhanced response to stimuli. Once activated by cytokines (IL-12, IL-15, IL-18), they produce more interferon-γ and GZMB. In previous studies, scRNA-seq was performed on both non-IL-2 stimulated and IL-2 stimulated NK cells, and it was found that GZMB was significantly upregulated after IL-2 stimulation, which was believed to be the enhanced-recall responses observed with in vitro differentiated CIML NK cells ^[5]^ .Compared with other clusters, these two clusters expressed high levels of inflammatory factors (S100B, S100A9, S100A2, S100A8) and keratin families (KRT14, KRT6A, KRT5), with lower expression levels of GZMA, PRF1, GZMB, and GNLY as other CD56^dim^ subsets. Further analysis showed that the expression levels of acute-phase response proteins (S100B, SAA1/SAA2, ferritin heavy chain 1 [FTH1]), heat shock protein family HSP90, and oxidative stress and inflammation regulators (PTGDS, DCD, E-cadherin ligand [ITGB7]) were higher in Cluster 16 than in Cluster 9 (Figure S6B). As PTGDS and S100B are important markers of CIML NK cell activation, so we deduced that Cluster 9 was the normal state of CIML NK cells, while Cluster 16 was CIML NK cells activated by cytokines in stress or inflammatory states.
